# Mental health interventions for climate change-induced disaster survivors in low- and middle-income countries (LMICs): A systematic review

**DOI:** 10.1017/gmh.2026.10249

**Published:** 2026-06-15

**Authors:** SeYeon Kim, Eszter Palócz, Jiwook Park, Seungwoo Kang

**Affiliations:** 1 Graduate School of Public Health, Seoul National University, Republic of Korea; 2 Institute of Psychology, Eötvös Loránd University, Hungary; 3 Clinton Health Access Initiative, Cambodia

**Keywords:** climate change, natural disasters, mental health, psychosocial interventions, LMICs

## Abstract

Climate change-induced disasters continue to disproportionately affect low, middle and upper-middle income countries (LMICs), often leaving survivors at risk of serious mental health challenges. Yet, mental health interventions in these settings remain limited and poorly integrated into health systems. This study explores the types and levels of existing mental health interventions for individuals affected by climate-induced disasters, using the Inter-Agency Standing Committee’s framework on Mental Health and Psychosocial Support. A systematic review was conducted following PRISMA guidelines across six databases, yielding 14 studies for final analysis. Key data on study design, disaster type, mental health conditions, intervention types and service providers were extracted and mapped against the Inter-Agency Standing Committee framework. While interventions were identified across all four layers of support, most were concentrated at the individual level, focusing on non-specialised services delivered by trained non-specialist providers (e.g., community health workers or lay counsellors) and specialised services provided by licenced clinical professionals, with limited emphasis on community-based or systems-level approaches. Results indicate such a significant gap, about 79% of the studies focused on the individual level, in mental health interventions in LMICs. It is therefore crucial to address these gaps to ensure comprehensive mental health care is available in disaster-prone countries.

## Impact statement

Climate change is driving a rapid rise in extreme weather events, placing disproportionate psychological and social burdens on affected low, middle and upper-middle income countries (LMICS and UMICs). This systematic review provides the first climate-specific synthesis of mental health and psychosocial support (MHPSS) interventions for survivors of climate-induced disasters in LMICs and UMICs, mapped against the Inter-Agency Standing Committee (IASC) framework. By analysing 14 peer-reviewed studies across floods and typhoons, the review highlights a critical imbalance in current mental health responses: interventions overwhelmingly target individual symptoms through specialised or focused non-specialised care, while community-based and structural support, fundamental for long-term resilience, remain largely absent or unevaluated. The review identifies clear evidence gaps, including minimal government-led MHPSS action, limited evaluation of community-level interventions and a near-complete absence of studies addressing other climate hazards such as droughts, heatwaves and wildfires. These gaps persist despite growing recognition that repeated climate shocks heighten risks of depression, anxiety, Post-Traumatic Stress Disorder (PTSD) and prolonged psychosocial distress – especially among populations facing poverty, displacement and fragile health systems. By systematically classifying interventions through the IASC pyramid, this study provides a structured evidence base to guide policymakers, Non-Governmental Organisations (NGOs) and disaster-management authorities in designing scalable, context-specific mental health strategies. The findings underscore the urgent need to integrate MHPSS into national climate adaptation and disaster risk reduction frameworks, expand task-sharing models to strengthen the workforce and invest in community-based and preventive approaches that address both immediate distress and long-term recovery. Ultimately, this review advances global understanding of how climate-driven disasters affect mental health in LMICs and UMICs; it offers actionable insights to build more resilient, equitable and sustainable psychosocial support systems as climate risks intensify.

## Background

The effects of climate change are increasingly becoming a major factor behind extreme weather events and environmental harm, with a pronounced impact on low- and middle-income countries (LMICs) (IPCC, 2021). LMICs face one of the most severe impacts of climate change due to their geographical locations, limited economic resources and capacity to cope with climate change-induced catastrophes like floods, droughts, cyclones and heat waves, which continue to intensify under global warming (IPCC, 2021; World Bank, 2021). Real-world examples illustrate the scale of this burden. The 2022 monsoon floods in Bangladesh displaced over 7 million people (United Nations Bangladesh, 2022, July), while Typhoon Haiyan in the Philippines caused extensive destruction and long-lasting psychosocial impacts across affected communities (Hugelius et al., 2017). Climate change continues to be labelled as one of the most significant global health challenges in the 21st century, impacting physical and psychological health in many direct and indirect ways. Direct effects include physical injury or loss of life due to exposure to extreme weather events such as floods, typhoons, heatwaves, droughts and storms, as well as the impact of rising sea levels, while indirect effects involve the spread of vector-borne and respiratory diseases, food and water insecurity, undernutrition, loss of livelihood and the breakdown of communities and forced displacement (IFRC, 2024; PAHO, 2024).

In recent years, the frequency and severity of climate-induced disasters have taken a serious toll on people’s mental health (IPCC, 2021). Although research shows that the majority of people exposed to disasters demonstrate psychological resilience and do not develop enduring mental health difficulties (Bonanno et al., 2010), the impact of such events can vary considerably for a significant proportion of the population, from minor distress to depression, anxiety, PTSD, sleep disorders and even suicide (Charlson et al., 2021). Evidence from global agencies reinforces this trend: the World Health Organisation (WHO) and the Intergovernmental Panel on Climate Change (IPCC) report that the psychological consequences of climate events intensify as disasters occur more frequently and affect larger populations. LMICs like Bangladesh, India, Thailand and Nigeria have faced catastrophic climate-induced disasters such as floods and landslides, which caused severe casualties and countless lives lost (Mahmud, 2024; Princewill, 2024; Wright et al., 2024). Floods are the most common, accounting for 47% of all natural disasters between 1995 and 2015- affecting more than 2.3 billion people globally (CRED, 2015). The increasing intensity and frequency of floods, compounded by growing populations and urbanisation in vulnerable areas, further amplify risks and impact on mental health (Stanke et al., 2012; Cianconi et al., 2020). This has led to mental health being one of the major contributors to the overall total burden of disease in LMICs (Rathod et al., 2017). Many countries, especially LMICs, face substantial gaps between mental health needs and the availability of appropriate services and support systems (WHO, 2024). In 2010, global losses due to mental, neurological and substance use disorders were estimated at US$2.5–8.5 trillion, depending on the assessment method used. Without a coordinated global response, the number of affected individuals is expected to nearly double by 2030. In response, the UN has prioritised mental health in its 2015–2030 Sustainable Development Goals (Chisholm et al., 2016). However, mental health services in many LMICs remain limited and poorly integrated, leaving those affected by climate disasters vulnerable to conditions like anxiety, depression and PTSD; often worsened by displacement, livelihood loss and social disruption (Berry et al., 20 10; World Bank, 2021). Mental health services, particularly at community and primary care levels, are largely absent in many LMICs. This gap stems from health systems historically prioritising clinical functions over psychosocial and supportive care, further compounded by limited financing, fragmented governance and insufficient provider training (Bolton et al., 2023).

A growing body of review literature has examined the mental health consequences of climate change or natural disasters; however, important gaps remain. Recent reviews such as Xue et al. (2024) summarised global MHPSS responses to climate change, while systematic reviews and meta-analyses by Brown et al. (2017), İkican et al. (2023) and Kip et al. (2025) evaluated psychosocial interventions for survivors of natural disasters more broadly. Additionally, the Cochrane review by Papola et al. (2020) synthesised evidence on mental disorder prevention among populations in humanitarian crises in LMICs. While these reviews offer valuable insights, most combine climate-related and non-climate-related disasters or focus broadly on humanitarian crises. None specifically examines mental health interventions for climate change-induced disasters through the structured lens of the IASC framework, nor do they compare intervention types across LMIC contexts or identify gaps in climate-specific MHPSS delivery. This creates a clear need for a targeted and up-to-date review that isolates climate-induced disasters and maps mental health interventions to a globally recognised system of care. Recent studies have highlighted significant evidence gaps on both mental health impact and effectiveness of interventions in LMICs (Charlson et al., 2021; Xue et al., 2024). Given these challenges, the IASC framework becomes particularly relevant. The framework outlines a layered system of mental health and psychosocial support following disasters – from basic services and community support to specialised clinical care – thereby offering a structured lens through which to assess the types of interventions that are being implemented and where gaps persist.

Global frameworks such as the SDGs and IASC guidelines are increasingly adapted by local governments and NGOs to guide climate-responsive mental health programming. For instance, the Bangladesh Ministry of Disaster Management and Relief has incorporated MHPSS considerations into its Standing Orders on Disaster and Cyclone Preparedness Programme (CPP), aligning national disaster actions with the Sendai Framework and SDG targets (Government of Bangladesh, 2019; Membele et al., 2022). Similarly, the Philippines integrates MHPSS and IASC guidelines into its National Disaster Risk Reduction and Management (NDRRM) Plan, operationalised at local levels through community-based safe spaces, psychosocial first-aid teams and NGO-led resilience activities (NDRRMC, 2020; Tolentino et al., 2021). Local NGOs such as BRAC, the Philippine Red Cross and Médecins Sans Frontières also routinely apply IASC MHPSS layers through mobile outreach, child-friendly spaces and community-led recovery programmes (IFRC, 2020; BRAC, 2021). These examples demonstrate how global frameworks are translated into context-specific strategies that support the mental health needs of climate-affected populations in LMICs. Given the increasing frequency of climate-induced disasters, there is an urgent need for evidence-based mental health interventions tailored to the specific needs of the disaster survivors in LMICs (World Bank, 2021; Charlson et al., 2022; Rowe and Nadkarni, 2024; UNEP, 2024).

This study, therefore, aims to fill the current evidence gap in mental health interventions for survivors of climate change-induced disasters in LMICs by analysing the mental health interventions available for climate change-induced disasters based on the Inter-Agency Standing Committee’s (IASC) framework. The findings are expected to contribute to the planning and implementation of responsive strategies and ultimately improve mental health outcomes and well-being for vulnerable populations in LMICs.

## Methodology

### Study design

**Figure 1.**
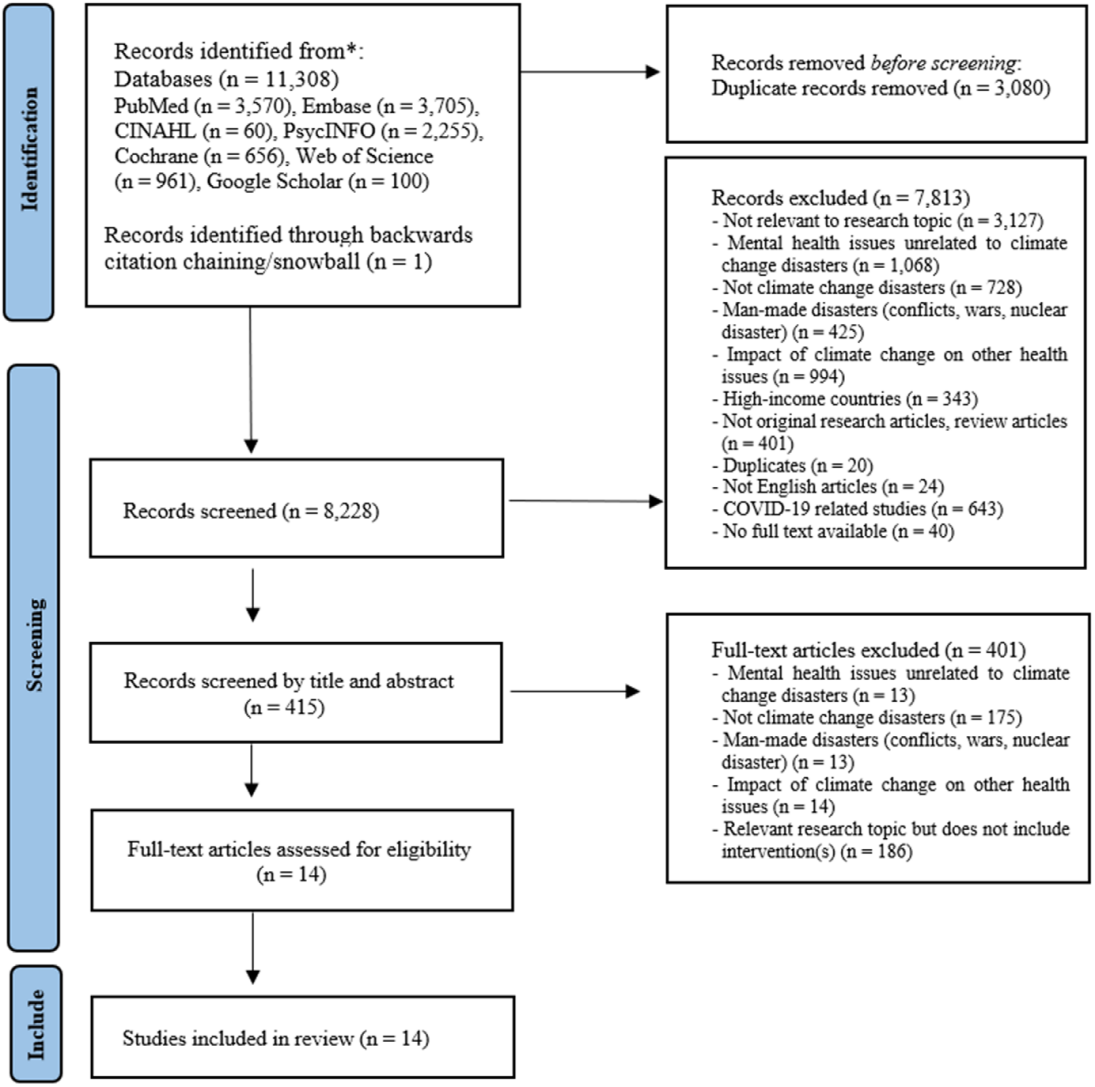
PRISMA flowchart of study selection.

This study employed a systematic review across six global bibliographic databases – Medline, Embase, PsycINFO, Cochrane Library, CINAHL and Web of Science – selected for their complementary strengths in capturing clinical, psychological, public health and disaster-related research. These databases offer broad international coverage and are particularly suited for identifying mental health interventions, behavioural health research and health-system responses relevant to LMICs, thereby aligning with the study’s focus on the intersection of climate-induced disasters and mental health. PsycINFO and CINAHL were specifically included to capture psychosocial, nursing and community-level interventions implemented in LMIC settings, while Medline, Embase and Cochrane provided access to biomedical, clinical and evidence-based intervention studies. Web of Science ensured coverage of interdisciplinary and environmental health research, which is essential for understanding climate-related mental health impacts. Specialised LMIC-focused or regional databases such as LILACS and African Journals Online (AJOL) were considered but not included due to significant overlap with the selected databases and limited indexing of mental health intervention studies related to climate-induced disasters. Google Scholar was included as a supplementary search tool to capture studies from LMICs that may not be indexed in major databases but still appear in peer-reviewed journals. Only peer-reviewed articles were extracted from Google Scholar search results; grey literature, theses, reports and nonpeer-reviewed documents were intentionally excluded despite appearing in search results. The “100 hits” refers specifically to the first 100 peer-reviewed entries returned for the search string, which Google Scholar ranks by relevance. This approach increases sensitivity to LMIC-published journal articles without compromising the exclusion of grey literature. The review followed the Preferred Reporting Items for Systematic Reviews and Meta-analyses (PRISMA- refer to Figure 1. for details) guidelines (Page et al., 2021) to ensure a transparent, systematic and replicable approach, improving the methodological rigour, reliability and consistency of the review process. The review protocol was registered a priori on PROSPERO (registration number: CRD42023456582).

### Search terms, eligibility criteria and quality appraisal

Search terms were classified into four categories (refer to Table 1 and Supplementary Appendix 1 for detail): (1) climate change-induced disasters, (2) mental health, (3) interventions and (4) LMICs. To address concerns about classification, the authors established explicit criteria for what constitutes a “climate change-induced disaster.” Climate-induced disasters were defined as those strongly linked in the scientific literature to anthropogenic climate change, primarily extreme weather events such as cyclonic storms, floods, landslides triggered by rainfall variability, tornadoes, wildfires and extreme heat. Disasters driven by geophysical processes (e.g., tectonic activity, volcanic eruptions and earthquakes) were excluded because they are not considered climate-driven according to IPCC and WHO frameworks. When ambiguity existed (e.g., landslides or flash floods), inclusion required documentation of meteorological drivers.

**Table 1.** Search terms Table 1. long description.

Line	Search terms
1	“Natural disaster*” or “Cyclonic storm*” or flood* or landslide* or tornadoe* or wildfire* or weather or “extreme weather” or “climate change*” or disaster* or “weather event*” or “extreme weather” or “global warming” or “climatic variability” or “cold wave*” or “heat wave*” or storm* or thunderstorm* or lightning or rain* or tornado* or avalanche* or drought* or “forest fire*” or “glacial lake outburst*”
2	Countries classified as LMICs according to the World Bank (2023) were identified and listed alongside the corresponding MeSH terms (refer to Supplementary Appendix 1 for the complete list)
3	”mental health" or “mental illness*” or “mental disorder*” or “psychological disorder*” or “psychiatric disorder*” or psychopatholog* or anxiet* or depress* or “post-traumatic stress” or “posttraumatic stress” or PTSD or suicide or “self harm” or “substance use” or “substance abuse” or “alcohol use” or “alcohol abuse” or “drug use” or “drug abuse” or “mood disorder*” or “anxiety disorder*” or anxiety or depression or “substance-related disorder*” or “prescription drug misuse” or “stress disorder*” or “traumatic” or “suicide”
4	“Psychotherapy” OR “Cognitive behavioral therapy” OR “Behavior* therapy” OR “Dialectical behavior therapy” OR “Mindfulness-based cognitive therapy” OR “Psychodynamic psychotherapy” OR “Cognitive therapy” OR “Family counseling” OR “Exposure therapy” OR “Group therapy” OR “Psychoeducation” OR “Counseling psychology” OR “Eye movement desensitization and reprocessing” OR “Medical diagnosis” OR “Psychological intervention” OR “Interpersonal psychotherapy” OR “Crisis intervention” OR “Solution-focused brief therapy” OR “Art therapy” OR “Behavior” OR “Therapy” OR “Antidepressant” OR “Mindfulness” OR “Trauma focused cognitive behavioral therapy” OR “intervention*” OR “program*” OR “project*” OR “health policy” OR “health policies” OR “health promotion”
5	#1 AND #2 AND #3 AND #4
	5 not ((exp animals/ or exp. invertebrates/ or animal experiments/ or animal models/ or exp. plants/ or exp. fungi/) not exp. humans/)

This study adopted the 2023 World Bank income classification to categorise LMICs. Mental health outcomes were defined broadly to align with global MHPSS guidance and to address the reviewer’s concern regarding scope. Eligible outcomes included (a) clinically defined mental disorders (e.g., PTSD, depression, anxiety), (b) subclinical psychological distress, (c) prevention of onset of mental disorders and (d) interventions aimed at improving psychological well-being among disaster-exposed populations. Thus, both treatment-focused and preventive interventions were eligible. Interventions were defined as any strategy or policy aimed at preventing or improving these outcomes, including psychotherapy, cognitive-behavioural therapies, mindfulness-based approaches, health-promotion programmes and broader health policies. No restrictions were placed on study design; qualitative, quantitative and mixed-methods studies were all eligible. This inclusive approach ensured that diverse forms of evidence, particularly common in LMIC settings where randomised controlled trials (RCTs) are less prevalent, were captured.

Each study identified through database search was independently screened by two authors after removal of duplicates. The review followed PRISMA guidelines (Page et al., 2021), which were selected because they enhance methodological transparency, improve the reliability and consistency of systematic reviews and establish clear reporting standards essential for minimising bias. Due to language and resource limitations, only articles written in English were included. This language restriction may have excluded relevant evidence from non-English-speaking LMIC regions; however, English-language inclusion was necessary to ensure feasible and consistent appraisal by all reviewers. Non-peer-reviewed or grey literature was acknowledged as potentially valuable but was excluded to maintain methodological rigour and comparability across studies.

The publication window (2000–2024) was selected to reflect a global shift in recognising the climate-disaster link following the IPCC’s Third Assessment Report (2021) and the development of standardised MHPSS frameworks such as the Inter-Agency Standing Committee (IASC) (2007). Literature that was published before this period was excluded as it generally examined natural disasters without linking them to climate change or contemporary psychosocial frameworks. Inclusion criteria consisted of (a) studies conducted in LMICs, (b) original research, (c) published between 2000 and 2024 and (d) focused on mental health or psychosocial interventions following climate-induced disasters. Importantly, interventions identified in previous reviews (e.g., psychological first aid, community-based art therapy, group-based cognitive behavioural therapy (CBT), school-based programmes) were not excluded a priori; rather, their absence in the final sample reflects the limited availability of peer-reviewed LMIC-specific studies meeting the climate-induced disaster definition. These further underscore the need for clearer documentation and evaluation of MHPSS interventions in LMIC settings. All studies meeting eligibility criteria (*n* = 14) underwent quality appraisal using the Mixed Methods Appraisal Tool (MMAT; see Supplementary Appendix 2). MMAT allows consistent evaluation across quantitative, qualitative and mixed-methods designs using design-specific criteria. Each study was independently assessed by two authors, with disagreements resolved through discussion and third-author adjudication. No studies were excluded during quality appraisal. Methodological limitations were considered in the narrative synthesis rather than used as exclusion criteria.

The final 14 studies were evaluated using the MMAT. MMAT is designed to appraise a wide range of methodological designs consistently, including quantitative, qualitative and mixed-methods research. It applies design-specific criteria assessing research questions, sampling strategy, representativeness, randomisation, data collection and measurement quality. Each study was independently assessed by two authors according to the criteria relevant to its design. Disagreements during the quality appraisal process were cross-checked and adjudicated by a third author, and consensus was reached through discussion, strengthening the reliability of the appraisal process. A total of seven criteria were assessed, each rated “Yes,” “No” or “Can’t tell.” The appraisal process is documented in Supplementary Appendix 2. Data synthesis followed a narrative and thematic approach as the heterogeneity of interventions, study designs and outcome measures made meta-analysis inappropriate. Studies were categorised by intervention type, delivery level (IASC layer) and mental health outcomes. Patterns, thematic trends and gaps were identified across studies to provide an integrated understanding of the evidence landscape.

Following the PRISMA flowchart, a total of 8,228 records were screened. Fourteen studies met the eligibility criteria and underwent MMAT quality appraisal, with no additional exclusions at this stage; therefore, 14 studies were included in the final review. This reflects the application of stringent eligibility criteria, particularly the focus on climate change–induced disasters. Consequently, a substantial number of studies – especially those related to geophysical hazards such as earthquakes – were excluded, as they fall outside the conceptual scope of climate-driven disasters. While these criteria may have reduced the breadth of the included evidence, they enabled a more focused and conceptually coherent synthesis aligned with the review objectives. By limiting the scope to climate-related hazards, this review provides more targeted insights into pathways and impacts directly associated with climate change, thereby strengthening the analytical specificity and policy relevance of the findings. Nevertheless, the conclusions should be interpreted in light of the defined scope of inclusion.

### Analysis framework

The Inter-Agency Standing Committee (IASC) (2007) for Mental Health and Psychosocial Support (MHPSS) framework addresses the mental health and psychosocial needs in emergency settings (IASC, 2012). It emphasises a multilayered approach that covers a different range of supports, from basic services to specialised clinical mental care.

**Figure 2.**
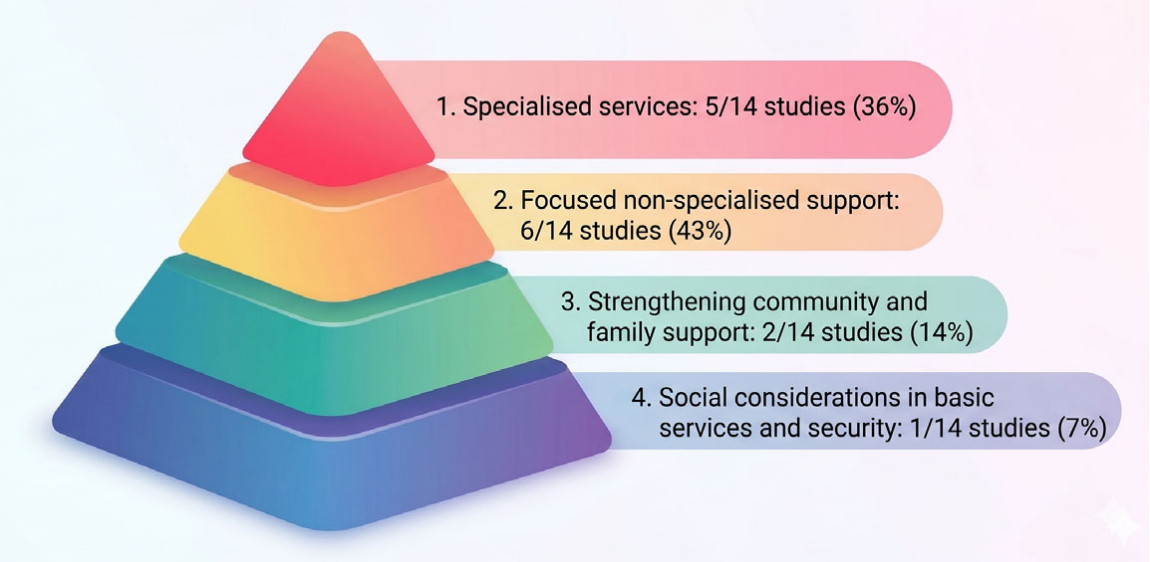
IASC intervention pyramid for MHPSS in emergencies (IASC, 2012 **).**

As illustrated by Figure 2, all layers of the intervention pyramid are essential and are ideally implemented simultaneously. Layer 4, at the bottom of the pyramid, focuses on providing basic services and security and ensuring that the well-being of affected populations is protected. Illustrative examples of interventions falling into layer 4 are essential services that meet basic physical needs such as shelter, food, water and basic health care, as well as considering the emotional and cultural impact the delivery of these services may have.

Layer 3 targets the strengthening of community and family support, as these are often disrupted during emergencies by loss, displacement, family separation and fear. Support focuses on rebuilding social ties and resilience through practical interventions. Examples include family reunification, community activities, parenting support, education or livelihood programmes and community groups such as women’s or youth networks.

Interventions in Layer 2 support a smaller group of affected individuals who require targeted, person-to-person support delivered by non-specialist providers. These services address more specific needs while remaining accessible within community or primary care settings. Examples include psychological first aid, basic psychosocial counselling and guided self-help.

Layer 1 refers to specialised services delivered by trained clinical professionals to address severe level of distress and mental health disorders. This level of support is typically needed by a smaller proportion of the population. Examples include the administration of medications and specialised therapeutic interventions, such as Trauma-Focused Cognitive Behavioural Therapy.

This study has adopted the IASC MHPSS framework to categorise and assess the different types of mental health interventions implemented to the survivors of climate-induced disasters in LMICs. It explored which layer(s) of the framework were most frequently addressed by existing interventions and which layer(s) are comparatively lacking. It therefore aimed to provide a clearer picture of how mental health interventions have been structured in disaster settings and where the gaps remain (IASC, 2007, 2012).

The types of mental health interventions and the service providers (who delivered the services) were extracted from each study and classified accordingly. Each study was categorised according to the IASC in Emergencies based on the type and level of intervention provided, while the service provider’s level of training was considered as a supporting indicator for classification.

## Results

### Study characteristics

A total of 14 studies were included in this review. Most studies focused on low- and middle-income countries (LMICs) (*n* = 9), followed by upper-middle income countries (UMICs) (*n* = 4) and low-income countries (LICs) (*n* = 1). The climate-induced natural disasters were predominantly floods (*n* = 11) and typhoons (*n* = 3). According to WHO’s regional distribution, Western Pacific region had the highest concentration of studies (*n* = 5), including two in the Philippines and one in China, Indonesia and Tuvalu, respectively. Regarding methodological design, the review identified four RCTs, five non-randomised studies and five descriptive or quasi-experimental studies. Detailed summary of study characteristics is provided in Table 2.

**Table 2.** Summary characteristics of included studies (*N* = 14) Table 2. long description.

Characteristics	*n* (%)
Income level (World Bank, 2023)	
Low-income countries (LIC)	1 (7.1)
Lower-middle-income countries (LMIC)	9 (64.3)
Upper- middle-income countries (UMIC)	4 (28.6)
Types of natural disasters	
Flood	11 (78.6)
Typhoon	3 (21.4)
WHO region (countries)	
Western Pacific (China, Indonesia, Philippines, Tuvalu)	5 (35.7)
Americas (Argentina, Haiti, Peru)	3 (21.4)
African (Burundi, Nigeria)	3 (21.4)
South-East Asia (India, Nepal)	2 (14.3)
Eastern Mediterranean (Pakistan)	1 (7.2)
Study design	# (%)
Quantitative non-randomised studies	5 (35.7)
Quantitative randomised controlled trials	4 (28.6)
Quantitative descriptive studies	4 (28.6)
Quasi-experimental designs	1 (7.1)
Publication year	# (%)
2020–2023	8 (57.1)
2011–2019	5 (35.7)
2000–2010	1 (7.1)

Table 3 summarises various characteristics of 14 studies, such as types of mental health disorders, interventions and components of care. A wide range of psychological symptoms among disaster survivors was identified. Considering psychological comorbidity in disasters, a total of 23 mental health disorders were identified. They are categorised into the three most common mental health disorders: depression (*n* = 8), PTSD (*n* = 8) and anxiety (*n* = 7). Regardless of the country’s income level or geographic region, survivors frequently presented with more than one mental health disorder, highlighting a high prevalence of comorbidity. Flood-related studies reported a broader distribution of depression and anxiety, whereas typhoon-related studies showed higher PTSD prevalence. Regional patterns also emerged. South Asian studies (India and Nepal) showed anxiety and depression, while studies from the Philippines presented PTSD-dominant profiles. In addition to these primary outcomes, several studies also assessed secondary outcomes such as coping skills, perceived stress, functioning and overall well-being, though these outcomes were not consistently measured across studies.

**Table 3.** Key characteristics of included studies Table 3. long description.

	References	Study design	Country (region/income level)	Type of natural disasters (year of disaster occurrence)	Disaster–research interval	Mental health disorders (anxiety, depression, PTSD)	Mental health interventions	Category of service provider (NGO/ Government /Research team)	Service Provider -Who delivered the service (Intervention)	Components of care
1	Adúriz et al. (2009)	Quantitative NRS	Argentina (Latin America /UMIC)	Flood (2003)	3 years	PTSD	Eye Movement Desensitisation and Reprocessing Integrative Group Treatment Protocol (EMDR-IGTP)	NGO	Argentine Society of Psychotrauma- Clinicians	1 Specialised services
2	Amin et al. (2020)	Quantitative RCT (Purposive sampling)	Pakistan (South Asia /LMIC)	Flood (2013)	7 years	PTSD	School-based intervention: Support for Students Exposed to Trauma (SSET) programme	Research team	University (psychology)- Clinically trained providers	1 Specialised services
3	Budiarto et al. (2019)	Quantitative NRS	Indonesia (Southeast Asia/LMIC)	Floods (2018)	1 year	ALL	Family psychoeducation therapy	Research team	Faculty of Nursing, Indonesia	2 Focused non-specialised support
4	Contreras et al. (2018)	Quantitative descriptive studies	Peru (Latin America /UMIC)	Flood and mudslides (2017)	1 year	Depression	“Mental health fair”	NGO	Socios En Salud (SES) - Trained community health workers (CHWs) and volunteers	2 Focused non-specialised support
5	Crombach and Siehl (2018)	Quantitative NRS	Burundi (sub-Saharan Africa/LIC)	Flood (2014)	4 years	Depression, PTSD	Narrative Exposure Therapy (NET)	NGO	Burundian Red Cross	2 Focused non-specialised support
6	Ede et al. (2022)	Quantitative RCT	Nigeria (sub-Saharan Africa/LMIC)	Flood (2019)	3 years	Depression	Rational Emotive Behaviour Therapy (REBT)	Research team	Psychotherapists	1 Specialised services
7	Gibson et al. (2021)	Quantitative NRS	Tuvalu (Western Pacific/UMIC*)	Typhoon (tropical cyclone) (2015)	6 years	PTSD	Skills for Life Adjustment and Resilience (SOLAR) programme adapted to Tuvalu culture	NGO	Tuvalu Association of NGO and Congregational Christian Church of Tuvalu	2 Focused non-specialised support
8	James et al. (2020)	Quantitative RCT	Haiti (Latin America and the Caribbean /LICs)	Flood (2010)	10 years	ALL	Integrated disaster preparedness group intervention	Research team	Each participant was facilitated by two trained Haitian lay mental health workers.	2 Focused non-specialised support
9	Jordans et al. (2021)	Quantitative RCT	Nepal (South Asia/LMIC)	Flood (2018)	3 years	Depression, PTSD	Group Problem Management Plus (PM+)	NGO	Transcultural Psychosocial Organisation (TPO)	2 Focused non-specialised support
10	Mathew (2021)	Quantitative NRS	India (South Asia/LMIC)	Flood (2018)	3 years	Anxiety	Yoga programme (breathing, meditation)	Research team	Kalari Rasayana Ayurveda Hospital, Kollam, Kerala	3 Strengthening community and family support
11	Ugwuoke et al. (2023)	Quantitative RCT	Nigeria (sub-Saharan Africa /LMIC)	Flood (2022)	1 year	Anxiety	Rational emotive family health therapy (REFHT)	Research team	University of Nigeria- Therapists	1 Specialised services
12	Waelde et al. (2018)	Quantitative NRS longitudinal	Philippines (Southeast Asia/LMIC)	Typhoon (2013)	5 years	ALL	4-h workshop, followed by an 8-week home study programme	Research team	School of Psychology, Palo Alto University, USA Department of Psychology, Ateneo de Manila University, Philippines	3 Strengthening community and family support
13	Weintraub et al. (2016)	Quantitative descriptive studies	Philippines (Southeast Asia/LMIC)	Typhoon (2013)	3 years	ALL	Psychoeducation, group discussion sessions, individual counselling/mental health care	NGO	Doctors without borders (MSF)-Expatriate psychologists	1 Specialised services
14	Zhong et al. (2020)	Quantitative NRS	China (Western Pacific/UMIC)	Flood (2016)	4 years	ALL	Living in planned shelters	Government	Chinese government	4 Social considerations in basic services and security

* Abbreviations: LIC, low-income countries; LMICs, lower-middle-income countries; UMICs, upper-middle income countries; RCT, randomised controlled trial; NRS, non-randomised studies.

Interventions were categorised according to the four layers of the IASC MHPSS pyramid as detailed in Table 3. Layer 1 (specialised services) accounted for 35.7% (*n* = 5) of the interventions. They are primarily involved clinical approaches such as the Eye Movement Desensitisation and Reprocessing (EMDR) therapy in Argentina and Rational Emotive Behaviour Therapy (REBT) in Nigeria delivered by trained psychologists or therapists. These programmes in layer 1 consistently reported reductions in PTSD, depression and trauma-related symptoms.

Layer 2 (non-specialised support) was the most common category (*n* = 6, 42.9%), utilising scalable protocols like “Skills for Life Adjustment and Resilience (SOLAR)” in Tuvalu and Problem Management Plus (PM+) in Nepal delivered by trained non-specialists or community workers. These programmes include interactive skills learning and management sessions between the service providers and the service receivers. Most of layer 2 studies reported a reduction in general distress, improvements in coping skills and increased functioning, though outcomes varied depending on training quality and fidelity of implementation.

In contrast to layers 1 and 2, interventions addressing the higher levels of the pyramid were less frequent. There were two studies (14.3%) in layer 3 (community and family support) and one study (7.1%) in layer 4 (social considerations in basic services and security). Mental health interventions/programmes in layer 3 are designed to strengthen social connections, ultimately fostering resilience and supporting mental well-being, such as yoga programmes in India and mindfulness-based home-study courses in the Philippines. Layer 3 interventions primarily demonstrated benefits for stress reduction, emotional regulation and community cohesion, though empirical evidence was limited by small samples and lack of control groups. Layer 4 involves a comprehensive programme in China integrating essential services in disaster recovery like provision of shelter and mental health needs.

The analysis of implementation models identified a significant gap in government-led initiatives. While the majority of interventions were implemented by NGOs (6 of 14 studies), only one intervention was formally led by a government body. The timing of these interventions also varied significantly relative to the disaster event. Acute-phase interventions that were delivered within weeks of the disaster primarily emphasised psychological first aid, psychoeducation or coping-skills training. Conversely, post-acute interventions, which occurred months or even years later, more frequently utilised structured psychotherapies such as CBT, REBT and EMDR.

There were notable regional patterns in the type of interventions as well. South Asia (e.g., India, Nepal, Bangladesh) demonstrated the greatest concentration of community-based or lay-facilitated interventions; Latin American studies (e.g., Argentina) primarily used clinically delivered psychotherapies; East and Southeast Asia (e.g., China, Philippines and Tuvalu) implemented structured group-based or shelter-based interventions. These differences reflect variations in health-system capacity, availability of trained personnel and disaster-response models.

Despite the clear psychological impact of climate change-related natural disasters, formal government-led mental health interventions in LMICs remain limited or undocumented. While the scarcity of published studies may indicate gaps in government action, it may also reflect the reality that existing initiatives have not been formally evaluated or captured through empirical research, resulting in limited visibility in the academic literature. Furthermore, NGO-led interventions generally demonstrated higher flexibility, clearer psychosocial components and stronger measured effectiveness compared with the single government-led intervention, although this disparity may also reflect publication bias towards NGO-operated programmes.

### Quality appraisal

The MMAT tool was used to critically appraise the 14 studies included. The studies included used quantitative randomised controlled trial, quantitative non-randomised and quantitative descriptive designs. Most studies satisfied the majority of MMAT criteria, indicating good methodological quality and clear alignment between research aims, data collection methods and analytical approaches. For quantitative randomised controlled trials, all MMAT criteria were fully met, including randomisation, group comparability, complete outcome data, assessor blinding and adherence to interventions. Among quantitative non-randomised studies, most studies satisfied the majority of criteria, though a few had limitations in participant representativeness (Adúriz et al., 2009; Mathew, 2021), confounder control and intervention adherence (Adúriz et al., 2009; Budiarto et al., 2019; Mathew, 2021). The majority of quantitative descriptive studies met MMAT criteria, with concerns about sampling relevance in Waelde et al. (2018). Overall, the studies reviewed demonstrated adequate quality and methodological rigour, supporting their inclusion in the synthesis.

### Intervention analysis

**Figure 3.**
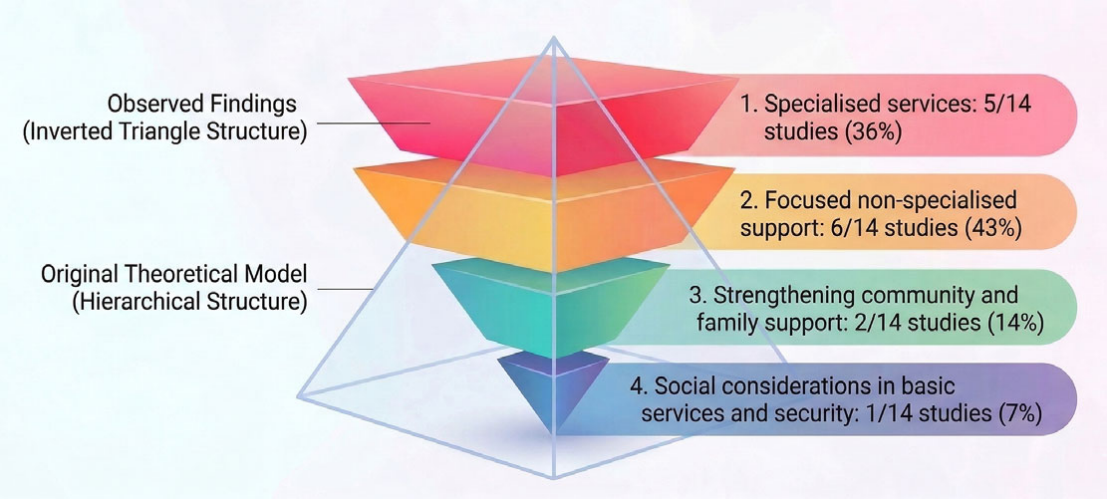
Distribution of included studies across the four layers of the IASC pyramid (*N* = 14).

A clear pattern emerged when examining the mental health interventions identified in this review: most interventions focused heavily on individual-level care, forming what resembles a top-heavy structure (inverted triangle) within the IASC pyramid framework (refer to Figure 3). While the IASC framework encourages a balanced, layered approach – starting from basic services and community support up to specialised care – most studies leaned towards interventions in the upper layers.

Out of the 14 studies included in the final review, 5 (36%) fell under Layer 1, where interventions were delivered by licenced mental health professionals – typically psychologists or psychiatrists – using approaches such as CBT, psychoeducation or trauma-focused techniques like EMDR. Six studies (43%) were categorised as Layer 2, involving trained non-specialist providers, most commonly community health workers or lay counsellors, who facilitated group-based psychoeducation, skill-building and basic problem-solving sessions.

Although this review distinguished Layers 1 and 2 based on whether the provider was a licenced professional or a trained non-specialist provider, this distinction is not without limitations. In practice, several interventions traditionally considered specialised psychological treatments may be delivered by trained lay providers following structured protocols and supervision. For example, Narrative Exposure Therapy (Crombach and Siehl, 2018) was originally developed as a specialised treatment for PTSD yet was delivered by trained non-specialist providers in the included study. In this review, such interventions were classified under Layer 2 despite their clinical complexity. This illustrates a conceptual overlap between Layers 1 and 2 and highlights the challenges of applying a strictly provider-based classification when specialised therapeutic approaches are task-shared to lay workers in humanitarian or low-resource settings. In contrast, only two studies addressed Layer 3, which focuses on strengthening family and community support systems through practices such as yoga, mindfulness and meditation (Waelde et al., 2018; Mathew, 2021) that foster both psychological relief and collective healing. Only one study was classified under Layer 4, where mental health support is integrated into basic services and safety; in Zhong et al. (2020), psychosocial support was delivered alongside emergency relief such as shelter, water, sanitation and primary healthcare. The overrepresentation of Layers 1 and 2 in the published literature does not necessarily indicate that higher-level basic services are absent in practice. Rather, Layer 4 interventions, designed to meet the broad needs of disaster-affected populations – are often not conceptualised or evaluated as “mental health interventions,” especially since most disaster-exposed individuals do not develop long-term psychological disorders. As a result, these foundational supports are less likely to appear in studies focused on clinical outcomes (e.g., PTSD, anxiety, depression), which may partly explain the limited number of Layer 4 studies identified in this review.

About 79% of the interventions identified were concentrated in Layers 1 and 2, reflecting the types of activities that are commonly published in peer-reviewed literature. This pattern should not be interpreted as evidence that community-level or structural resilience efforts are absent; rather, such interventions are often implemented through local government, NGOs and community networks but are rarely captured in English-academic journals, which creates a visibility gap. The dominance of individual-level interventions in published studies therefore reflects limitations in what is typically recorded and disseminated, not necessarily in what is occurring on the ground. Regarding effectiveness, EMDR, CBT, REBT, PM+ and SOLAR showed the strongest improvements across depression, anxiety and PTSD symptoms. Yoga and mindfulness demonstrated improvements in mental health, especially in stress reduction and emotional regulation (please to refer to Table 3. for detail). Even so, relying primarily on individually focused approaches, without corresponding documentation of community or structural strategies, risks overlooking the broader protective factors that support long-term recovery. Strengthening mental health responses requires engaging all levels of the IASC pyramid, pairing clinical care with community-based support and structural safety nets (WHO, 2022; Lund, 2023).

## Discussion

As climate change ramps up the frequency of extreme weather events like floods and typhoons, the mental health impact on those affected is becoming increasingly urgent (Hayes et al., 2018). This review sheds light on the growing need for mental health support in LMICs where these disasters are becoming more common. Even though Asia experienced the highest number of climate-induced disasters from 2000 to 2019, there is still a lack of research on how these events affect mental health (UNDRR, 2020; Our World in Data, 2024). Community-based psychosocial support has shown potential in alleviating distress during crises, but the focus on long-term mental health resilience, especially in high-risk areas, is still largely missing (Bansal and Arih, 2022; Barnett et al., 2023). These findings are broadly consistent with previous reviews on post-disaster mental health in LMICs, which similarly observed that interventions tend to rely on short-term psychological first aid rather than sustained, community-level or systems-level support (e.g., Tol et al., 2011; Haroz et al., 2020). However, this review additionally highlights that the imbalance is particularly pronounced in climate-related disasters, where multi-hazard exposure and repeated displacement demand more durable forms of support. It is crucial to weave Mental Health and Psychosocial Support (MHPSS) into national climate adaptation strategies to reduce lasting damage and foster recovery (UNDRR, 2020; WHO, 2022). Importantly, the mental health impacts of climate-induced disasters are often intensified by socioeconomic vulnerabilities – such as poverty, inadequate housing, weak healthcare infrastructure and social inequality – that limit people’s ability to prepare for, cope with and recover from crises (World Bank, 2021; WHO, 2022; IPCC, 2021). These intersecting vulnerabilities disproportionately affect marginalised populations, including low-income households, informal settlers and rural communities, who experience greater psychological strain and face structural barriers to accessing mental health support during and after disasters (Stanke et al., 2012; Lund et al., 2018). Understanding these interconnected challenges is critical to designing equitable and effective mental health responses in LMICs.

Current mental health interventions in LMICs are largely concentrated at the individual level, with comparatively limited attention given to community and structural approaches. According to the IASC Intervention Pyramid for MHPSS in Emergencies, greater emphasis should be placed on higher system-level layers, particularly community and family support and the integration of psychosocial considerations within basic services, which remain underrepresented in current practice. Community-based activities such as yoga, mindfulness and social bonding initiatives have shown potential to improve psychological well-being while simultaneously strengthening community resilience (Waelde et al., 2018; Mathew, 2021). However, the expansion of these community- and system-level interventions faces significant barriers in many LMICs, including limited government budgets, fragmented governance structures, shortages of trained personnel and persistent cultural stigma surrounding mental health, all of which may reduce programme uptake and long-term sustainability (Patel et al., 2018; Kola et al., 2021). As a result, even promising community-based initiatives often remain small-scale pilot programmes rather than becoming integrated components of national health systems.

Moreover, ensuring the quality and reach of services at community and system levels requires strengthened training and accreditation mechanisms for non-clinical mental health providers (Barnett et al., 2023). This issue is particularly relevant given ongoing debates regarding the effectiveness of low-intensity interventions, such as psychoeducation or mindfulness-based approaches, compared with high-intensity interventions, such as EMDR or CBT. While low-intensity interventions are generally more scalable and adaptable to diverse cultural contexts, evidence suggests that trauma-focused psychotherapy tends to produce stronger symptom reduction, particularly for PTSD, indicating the potential value of a stepped-care approach (Keshavan et al., 2020). Furthermore, mental health support should be systematically integrated into broader disaster preparedness and response mechanisms, including emergency shelters and essential service delivery. Such integration requires coordinated action among key stakeholders, including LMIC governments, international organisations and NGOs (IASC, 2007; WHO, 2022). Overall, the available evidence suggests that MHPSS interventions in LMICs remain heavily focused on addressing individual-level symptoms rather than strengthening community resilience or systemic support structures. Although reported intervention outcomes are generally positive, the heavy reliance on NGO-led delivery models and the limited availability of long-term follow-up evidence highlight the need for sustainable, government-integrated mental health strategies that can be embedded within national public health systems in disaster-prone LMICs.

Actionable policy recommendations emerging from this review include integrating MHPSS explicitly into national disaster risk reduction frameworks, allocating climate adaptation funds toward mental health services, embedding trained community mental health workers within emergency response systems and developing multi-sectoral protocols that link health, protection, and livelihood services. Given the resource constraints faced by many LMICs, practical approaches to implementing community-based interventions may include task-sharing models that train community members or volunteers as psychosocial first responders, embedding MHPSS activities into existing community groups (such as women’s associations or local health committees) and leveraging faith-based or cultural institutions as platforms for group-based support. Furthermore, the exclusion of other climate-induced disasters, such as droughts, heatwaves and wildfires, from the current evidence base represents a substantial limitation, as these events are increasing in frequency and may have distinct mental health consequences that are not captured in flood- and typhoon-focused research. Finally, several research gaps require urgent attention: the near absence of studies from low-income countries, limited evidence for Layer 3 and 4 interventions, a lack of studies on droughts, heatwaves and wildfires and minimal examination of high-risk groups such as older adults, people with disabilities and small-island communities. Addressing these gaps will be critical for building a more complete understanding of mental health resilience in a rapidly changing climate.

### Strengths and limitations

Although this study has strengths in highlighting mental health interventions available to populations in LMICs facing climate-induced challenges, the studies included in the final review focused only on floods and typhoons. Despite including “heatwaves” in the search terms, no relevant studies were identified, underscoring a significant gap not only in intervention implementation but also in research coverage within LMICs (CRED, 2015). The lack of studies on droughts and wildfires, two forms of climate-induced disasters with rising global incidence, further limits the generalisability of the findings and points to an urgent need for broader disaster-type representation in future research.

Furthermore, the evidence base remains limited by short follow-up periods, making it difficult to assess the long-term effects of the interventions reported. For data quality considerations, this review included only peer-reviewed original journal articles, meaning that potentially valuable insights from grey literature, programme reports or working papers may have been omitted. To build a more comprehensive understanding of mental health responses in LMICs, future studies should incorporate a broader range of evidence sources. In addition, the methodological limitations in this field are substantial; many studies employ small samples, lack control groups or rely on short-term outcomes, highlighting the need to include quality-assessment findings in future publications to better contextualise the strength of the existing evidence base. It is, therefore, important to acknowledge that only one study in this review was conducted in a low-income country, which limits the generalisability of the findings across all LMIC settings. Finally, several methodological limitations must be acknowledged: exclusion of non-English and grey literature, limited representation of low-income countries, variability in study design quality and the predominance of short-term outcomes limit the generalisability of findings. These constraints highlight the need for more rigorous, longitudinal and locally grounded research in LMICs.

## Conclusion

With the increasing frequency and intensity of climate change–induced disasters globally, the demand for effective mental health and psychosocial support (MHPSS) interventions for affected populations is growing. This review synthesised the available evidence on MHPSS interventions for survivors of climate-related disasters in LMICs and identified several important findings. First, the existing evidence base remains limited and highly concentrated on hydrometeorological disasters, with all included studies focusing on floods (*n* = 11) and typhoons (*n* = 3). Second, while a range of MHPSS interventions has been implemented, there is insufficient evidence on their effectiveness, scalability and long-term sustainability within LMIC contexts. Importantly, the concentration of evidence on floods and typhoons highlights a critical gap in the literature. Despite the inclusion of a broader range of climate hazards in the search strategy, no eligible studies were identified for droughts, heatwaves, wildfires or other climate-related events. Given that different types of climate hazards may involve distinct exposure pathways, stressors and mental health outcomes, the findings of this review should be interpreted with caution. In particular, generalising these results to MHPSS responses for other climate-related disasters may not be appropriate without further evidence.

Nevertheless, by applying a focused scope on climate change–induced disasters, this review provides targeted and policy-relevant insights into current intervention approaches and their limitations within LMIC settings. The findings underscore the need for more rigorous and context-sensitive research, particularly on underrepresented climate hazards, as well as stronger evaluation frameworks to assess intervention effectiveness. Future studies should prioritise expanding the evidence base across diverse disaster types and developing adaptable MHPSS models that can respond to the varying mental health profiles associated with different climate risks.

## Supporting information

10.1017/gmh.2026.10249.sm001 Kim et al. supplementary material Kim et al. supplementary material

## Data Availability

Appendix 1. Search Strategy(terms); Appendix 2. Mixed Methods Appraisal Tool (MMAT) table.

## Supplementary material

The supplementary material for this article can be found at http://doi.org/10.1017/gmh.2026.10249.

## Long descriptions

### Table 1. Long description

The table consists of two columns: Line and Search terms.

Line 1: Natural disaster or Cyclonic storm or flood or landslide or tornadoe or wildfire or weather or extreme weather or climate change or disaster or weather event or extreme weather or global warming or climatic variability or cold wave or heat wave or storm or thunderstorm or lightning or rain or tornado or avalanche or drought or forest fire or glacial lake outburst.

Line 2: Countries classified as L M I C s according to the World Bank 2021 were identified and listed alongside the corresponding M e S H terms.

Line 3: mental health or mental illness or mental disorder or psychological disorder or psychiatric disorder or psychopatholog or anxiet or depress or post-traumatic stress or posttraumatic stress or P T S D or suicide or self harm or substance use or substance abuse or alcohol use or alcohol abuse or drug use or drug abuse or mood disorder or anxiety disorder or anxiety or depression or substance-related disorder or prescription drug misuse or stress disorder or traumatic or suicide.

Line 4: Psychotherapy or Cognitive behavioral therapy or Behavior therapy or Dialectical behavior therapy or Mindfulness-based cognitive therapy or Psychodynamic psychotherapy or Cognitive therapy or Family counseling or Exposure therapy or Group therapy or Psychoeducation or Counseling psychology or Eye movement desensitization and reprocessing or Medical diagnosis or Psychological intervention or Interpersonal psychotherapy or Crisis intervention or Solution-focused brief therapy or Art therapy or Behavior or Therapy or Antidepressant or Mindfulness or Trauma focused cognitive behavioral therapy or intervention or program or project or health policy or health policies or health promotion.

Line 5: #1 AND #2 AND #3 AND #4.

Final row: 5 not ((exp animals/ or exp. invertebrates/ or animal experiments/ or animal models/ or exp. plants/ or exp. fungi/) not exp. humans/).

Navigate back to Table 1..

### Table 2. Long description

The table is organized into two columns: Characteristics and n (%).

Income level (World Bank, 2021):

* Low-income (L I C): 1 (7.1%)

* Lower-middle (L M I C): 9 (64.3%)

* Upper-middle (U M I C): 4 (28.6%)

Types of natural disasters:

* Flood: 11 (78.6%)

* Typhoon: 3 (21.4%)

W H O region (countries):

* Western Pacific (China, Indonesia, Philippines, Tuvalu): 5 (35.7%)

* Americas (Argentina, Haiti, Peru): 3 (21.4%)

* African (Burundi, Nigeria): 3 (21.4%)

* South-East Asia (India, Nepal): 2 (14.3%)

* Eastern Mediterranean (Pakistan): 1 (7.2%)

Study design:

* Quantitative non-randomised studies: 5 (35.7%)

* Quantitative randomised controlled trials: 4 (28.6%)

* Quantitative descriptive studies: 4 (28.6%)

* Quasi-experimental designs: 1 (7.1%)

Publication year:

* 2020–2023: 8 (57.1%)

* 2011–2019: 5 (35.7%)

* 2000–2010: 1 (7.1%)

Navigate back to Table 2..

### Table 3. Long description

The table contains 11 columns: Reference, Study design, Country (region/income level), Natural disasters (year), Disaster-research interval, Mental health disorders (anxiety, depression, P T S D), Mental health interventions, Category of service provider, Who delivered the service, and Components of care.

Key entries include:

1. Adúriz et al. (2009): Quantitative N R S, Argentina, Flood (2003), 3 years interval, P T S D, E M D R-I G T P intervention, delivered by N G O clinicians, focused on Specialized services.

2. Amin et al. (2020): Quantitative R C T, Pakistan, Flood (2013), 7 years interval, P T S D, S S E T programme, delivered by Research team (University psychology), focused on Specialized services.

3. Budiarto et al. (2019): Quantitative N R S, Indonesia, Floods (2018), 1 year interval, All disorders, Family psychoeducation, delivered by Research team (Nursing), focused on Focused non-specialized support.

4. Contreras et al. (2018): Quantitative descriptive, Peru, Flood/mudslides (2017), 1 year interval, Depression, Mental health fair, delivered by N G O (Socios En Salud), focused on Focused non-specialized support.

5. Crombach and Siehl (2018): Quantitative N R S, Burundi, Flood (2014), 4 years interval, Depression and P T S D, Narrative Exposure Therapy, delivered by N G O (Red Cross), focused on Focused non-specialized support.

6. Ede et al. (2022): Quantitative R C T, Nigeria, Flood (2019), 3 years interval, Depression, R E B T intervention, delivered by Research team psychotherapists, focused on Specialized services.

7. Gibson et al. (2021): Quantitative N R S, Tuvalu, Typhoon (2015), 6 years interval, P T S D, S O L A R programme, delivered by N G O/Church, focused on Focused non-specialized support.

8. James et al. (2020): Quantitative R C T, Haiti, Flood (2010), 10 years interval, All disorders, Integrated disaster preparedness, delivered by Research team (lay workers), focused on Focused non-specialized support.

9. Jordans et al. (2021): Quantitative R C T, Nepal, Flood (2018), 3 years interval, Depression and P T S D, Group P M plus, delivered by N G O (T P O), focused on Focused non-specialized support.

10. Mathew (2021): Quantitative N R S, India, Flood (2018), 3 years interval, Anxiety, Yoga programme, delivered by Research team (Hospital), focused on Strengthening community and family support.

11. Ugwuoke et al. (2023): Quantitative R C T, Nigeria, Flood (2022), 1 year interval, Anxiety, R E F H T intervention, delivered by Research team (University), focused on Specialized services.

12. Waelde et al. (2018): Quantitative N R S longitudinal, Philippines, Typhoon (2013), 5 years interval, All disorders, Workshop/home study, delivered by Research team (Palo Alto/Ateneo Universities), focused on Strengthening community and family support.

13. Weintraub et al. (2016): Quantitative descriptive, Philippines, Typhoon (2013), 3 years interval, All disorders, Psychoeducation/counseling, delivered by N G O (M S F), focused on Specialized services.

14. Zhong et al. (2020): Quantitative N R S, China, Flood (2016), 4 years interval, All disorders, Planned shelters, delivered by Government, focused on Social considerations in basic services and security.

Navigate back to Table 3..
