# Supplementary material for: Mental health interventions for climate change-induced disaster survivors in low- and middle-income countries (LMICs): A systematic review
Source: Glob Ment Health (Camb). 2026 Jun 15;13:e154. doi: 10.1017/gmh.2026.10249 (PMC13375834; doi:10.1017/gmh.2026.10249)
Supplement: Kim et al. supplementary material [file S2054425126102490sup001.docx]

**Appendix 1. Search terms**

| **Line** | **Search terms** |
| --- | --- |
| 1 | “Natural disaster*” or “Cyclonic storm*” or flood* or landslide* or tornadoe* or wildfire* or weather or “extreme weather” or "climate change*" or disaster* or "weather event*" or "extreme weather" or "global warming" or "climatic variability" or "cold wave*" or "heat wave*" or storm* or thunderstorm* or lightning or rain* or tornado* or avalanche* or drought* or "forest fire*" or "glacial lake outburst*” |
| 2 | "Deprived Countries" OR "Deprived Population" OR "Deprived Populations" OR "Developing Countries" OR "Developing Country" OR "Developing Economies" OR "Developing Economy" OR "Developing Nation" OR "Developing Nations" OR "Developing Population" OR "Developing Populations" OR "Developing World" OR "LAMI Countries" OR "LAMI Country" OR "Less Developed Countries" OR "Less Developed Country" OR "Less Developed Economies " OR "Less Developed Nation" OR "Less Developed Nations" OR "Less Developed World" OR "Lesser Developed Countries" OR "Lesser Developed Nations" OR "LMIC" OR "LMICS" OR "Low GDP" OR "Low GNP" OR "Low Gross Domestic" OR "Low Gross National" OR "Low Income Countries" OR "Low Income Country" OR "Low Income Economies " OR "Low Income Economy" OR "Low Income Nations" OR "Low Income Population" OR "Low Income Populations" OR "Lower GDP" OR "lower gross domestic" OR "Lower Income Countries" OR "Lower Income Country" OR "Lower Income Nations" OR "Lower Income Population" OR "Lower Income Populations" OR "Middle Income Countries" OR "Middle Income Country" OR "Middle Income Economies " OR "Middle Income Nation" OR "Middle Income Nations" OR "Middle Income Population" OR "Middle Income Populations" OR "Poor Countries" OR "Poor Country" OR "Poor Economies"  OR "Poor Economy" OR "Poor Nation" OR "Poor Nations" OR "Poor Population" OR "Poor Populations" OR "poor world" OR "Poorer Countries" OR "Poorer Economies" OR "Poorer Economy" OR "Poorer Nations" OR "Poorer Population" OR "Poorer Populations" OR "Third World" OR "Transitional Countries" OR "Transitional Country" OR "Transitional Economies" OR "Transitional Economy" OR "Under Developed Countries" OR "Under Developed Country" OR "under developed nations" OR "Under Developed World" OR "Under Served Population" OR "Under Served Populations" OR "Underdeveloped Countries" OR "Underdeveloped Country" OR "underdeveloped economies" OR "underdeveloped nations" OR "underdeveloped population" OR "Underdeveloped World" OR "Underserved Countries" OR "Underserved Nations" OR "Underserved Population" OR "Underserved Populations" OR "low-income setting"  OR "low-income settings"  OR "low-income countries"  OR "middle-income setting"  OR "middle-income settings"  OR "middle-income countries"  OR "low income setting"  OR "low income settings"  OR "low income countries"  OR "middle income setting"  OR "middle income settings"  OR "middle income countries"  OR "transition economy"  OR "transition economies"  OR "economically disadvantaged"  OR "less favored areas"  OR "developing world"  OR "developing country"  OR "developing countries"  OR "under-developed country"  OR "under-developed countries"  OR "less-developed country"  OR "less-developed countries"  OR "less-developed nation"  OR "less-developed nations"  OR "third-world country"  OR "third-world countries"  OR Africa  OR African  OR Asia  OR Asian  OR "Latin America"  OR "South America"  OR Afghanistan or Albania or Algeria or Angola or Argentina or Armenia or Azerbaijan or Bangladesh or Belarus or Belize or Benin or Bhutan or Bolivia or "Bosnia and Herzegovina" or Botswana or Brazil or Bulgaria or "Burkina Faso" or Burundi or "Cabo Verde" or Cambodia or Cameroon or "Central African Republic" or Chad or China or Colombia or Comoros or Congo or "Costa Rica" or "Cote d'Ivoire" or Cuba or "Democratic Republic of the Congo" or “Republic of the Congo” or Djibouti or Dominica or "Dominican Republic" or "DR Congo" or Ecuador or Egypt or “Arab Republic of Egypt” or "El Salvador" or "Equatorial Guinea" or Eritrea or Eswatini or Ethiopia or Fiji or Gabon or Gambia or “The Gambia” or Georgia or Ghana or Grenada or Guatemala or Guinea or Guinea-Bissau or Haiti or Honduras or India or Indonesia or Iran or “Islamic Republic of Iran” or Iraq or Jamaica or Jordan or Kazakhstan or Kenya or Kiribati or "Democratic People's Republic of Korea" OR "North Korea" or Kosovo or “Kyrgyz Republic” or Kyrgyzstan or Laos or “Lao PDR” or Lebanon or Lesotho or Liberia or Libya or Macedonia or “North Macedonia” or Madagascar or Malawi or Malaysia or Maldives or Mali or "Marshall Islands" or Mauritania or Mauritius or Mexico or Micronesia or “Federated States of Micronesia” or Moldova or Mongolia or Montenegro or Morocco or Mozambique or Myanmar or Namibia or Nepal or Nicaragua or Niger or Nigeria or Pakistan or Palau or "Papua New Guinea" or Paraguay or Peru or Philippines or Russia or “Russian Federation” or Rwanda or "Saint Lucia" or "Saint Vincent and the Grenadines" or Samoa or "Sao Tome And Principe" or Senegal or Serbia or "Sierra Leone" or "Solomon Islands" or Somalia or "South Africa" or "South Sudan" or "Sri Lanka" or Sudan or Suriname or Swaziland or Syria or “Syrian Arab Republic” or Tajikistan or Tanzania or Thailand or Timor-Leste or Togo or Tonga or Tunisia or Turkey or Türkiye or Turkmenistan or Tuvalu or Uganda or Ukraine or Uzbekistan or Vanuatu or Vietnam or “West Bank and Gaza” or Yemen or Zambia or Zimbabwe or American Samoa OR Armenia OR Armenian OR Azerbaijan OR Republic Of Belarus OR Belarus OR Byelarus OR Belorussia OR Byelorussian OR British Honduras OR Benin OR Dahomey OR Bhutan OR Bolivia OR Herzegovina OR Bechuanaland OR Brasil OR Burkina Fasso OR Upper Volta OR Urundi OR Cape Verde OR Kampuchea OR Khmer Republic OR Cameron OR Cameroun OR Ubangi Shari OR Comoro Islands OR Iles Comores OR Mayotte OR Zaire OR Cote Divoire OR Cote D Ivoire OR Ivory Coast OR French Somaliland OR Spanish Guinea OR Gabonese Republic OR "Georgia (Republic)" OR Georgian OR Gold Coast OR British Guiana OR Hispaniola OR Timor OR Isle Of Man OR Kazakh OR North Korea OR Kirghizia OR Kirgizstan OR Kirghiz OR "Lao People's Democratic Republic" OR Lebanese Republic OR Basutoland OR Libyan Arab Jamahiriya OR "Macedonia (Republic)" OR Malagasy Republic OR Nyasaland OR Indian Ocean Islands OR Indian Ocean OR Northern Mariana Islands OR Moldovian OR Ifni OR Portuguese East Africa OR Burma OR Namibia OR Nepal OR Netherlands Antilles OR New Guinea OR Philipines OR Phillipines OR Phillippines OR Ruanda OR Pacific Islands OR Samoan Islands OR Navigator Island OR Navigator Islands OR Solomon Island OR Norfolk Island OR Norfolk Islands OR Ceylon OR "St. Lucia" OR Saint Vincent OR "St. Vincent" OR Grenadines OR Surinam OR Dutch Guiana OR Netherlands Guiana OR Syrian Arab Republic OR Tadjikistan OR Tadzhikistan OR Tadzhik OR Tanganyika OR Siam OR Timor Leste OR East Timor OR Togo OR Togolese Republic OR Tonga OR Tobago OR Tunisia OR "Turkey (Republic)" OR Turkmenistan OR Turkmen OR Uganda OR Ukraine OR Uzbekistan OR Uzbek OR Vanuatu OR New Hebrides OR Viet Nam OR Middle East OR West Bank OR Gaza OR Palestine OR Yemen OR Yugoslavia OR Zambia OR Zimbabwe OR Northern Rhodesia OR Global South OR “Africa South Of The Sahara” OR Sub-Saharan Africa OR Subsaharan Africa OR Africa, Central OR Central Africa OR Africa, Northern OR North Africa OR Northern Africa OR Magreb OR Maghrib OR Sahara OR Africa, Southern OR Southern Africa OR Africa, Eastern OR East Africa OR Eastern Africa OR Africa, Western OR West Africa OR Western Africa OR West Indies OR Indian Ocean Islands OR Caribbean OR Central America OR Latin America OR "South And Central America" OR South America OR Asia, Central OR Central Asia OR Asia, Northern OR North Asia OR Northern Asia OR Asia, Southeastern OR Southeastern Asia OR South Eastern Asia OR Southeast Asia OR South East Asia OR Asia, Western OR Western Asia |
| 3 | "mental health" or "mental illness*" or "mental disorder*" or "psychological disorder*" or "psychiatric disorder*" or psychopatholog* or anxiet* or depress* or "post-traumatic stress" or "posttraumatic stress" or PTSD or suicide or "self harm" or "substance use" or "substance abuse" or "alcohol use" or "alcohol abuse" or "drug use" or "drug abuse" or "mood disorder*" or "anxiety disorder*" or anxiety or depression or "substance-related disorder*" or "prescription drug misuse" or "stress disorder*" or "traumatic" or "suicide" |
| 4 | “Psychotherapy” OR “Cognitive behavioral therapy” OR “Behavior* therapy” OR “Dialectical behavior therapy” OR “Mindfulness-based cognitive therapy” OR “Psychodynamic psychotherapy” OR “Cognitive therapy” OR “Family counseling” OR “Exposure therapy” OR “Group therapy” OR “Psychoeducation” OR “Counseling psychology” OR “Eye movement desensitization and reprocessing” OR “Medical diagnosis” OR “Psychological intervention” OR “Interpersonal psychotherapy” OR “Crisis intervention” OR “Solution-focused brief therapy” OR “Art therapy” OR “Behavior” OR “Therapy” OR “Antidepressant” OR “Mindfulness” OR “Trauma focused cognitive behavioral therapy” OR “intervention*” OR “program*” OR “project*” OR “health policy” OR “health policies” OR “health promotion” |
| 5 | #1 AND #2 AND #3 AND #4 |
| 6 | 5 not ((exp animals/ or exp invertebrates/ or animal experiments/ or animal models/ or exp plants/ or exp fungi/) not exp humans/) |

**Appendix 2. Quality appraisal using the Mixed Methods Appraisal Tool (MMAT) (Page et al., 2021)**

| **No.** | **Author (year)** | **Screening questions** | | **Quantitative randomised controlled trials** | | | | |
| --- | --- | --- | --- | --- | --- | --- | --- | --- |
|  |  | S1. Are there clear research questions? | S2. Do the collected data allow to address the research questions? | 2.1. Is randomization appropriately performed? | 2.2. Are the groups comparable at baseline? | 2.3. Are there complete outcome data? | 2.4. Are outcome assessors blinded to the intervention provided? | 2.5. Did the participants adhere to the assigned intervention? |
| 1 | Amin et al. (2020) | Yes | Yes | Yes | Yes | Yes | Yes | Yes |
| 2 | James et al. (2020) | Yes | Yes | Yes | Yes | Yes | Yes | Yes |
| 3 | Jordans et al. (2021) | Yes | Yes | Yes | Yes | Yes | Yes | Yes |
| 4 | Ede et al. (2022) | Yes | Yes | Yes | Yes | Yes | Yes | Yes |
| 5 | Ugwoke et al. (2023) | Yes | Yes | Yes | Yes | Yes | Yes | Yes |
|  | **Author (year)** | **Screening questions** | | **Quantitative non-randomised studies** | | | | |
|  |  | S1. Are there clear research questions? | S2. Do the collected data allow to address the research questions? | 3.1. Are the participants representative of the target population? | 3.2. Are measurements appropriate regarding both the outcome and intervention (or exposure)? | 3.3. Are there complete outcome data? | 3.4. Are the confounders accounted for in the design and analysis? | 3.5. During the study period, is the intervention administered (or exposure occurred) as intended? |
| 1 | Crombach and Siehl (2018) | Yes | Yes | Yes | Yes | Yes | Yes | Yes |
| 2 | Budiarto et al. (2019) | Yes | Yes | Yes | Yes | Yes | No | No |
| 3 | Zhong et al. (2020) | Yes | Yes | Yes | Yes | Yes | Yes | Yes |
| 4 | Gibson et al. (2021) | Yes | Yes | Yes | Yes | Yes | Yes | Yes |
| 5 | Mathew (2021) | Yes | Yes | No | Yes | Yes | No | No |
| 6 | Aduriz et al. (2009) | Yes | Yes | Can’t tell | Yes | Yes | No | No |
|  | **Author (year)** | **Screening questions** | | **Quantitative descriptive studies** | | | | |
|  |  | S1. Are there clear research questions? | S2. Do the collected data allow to address the research questions? | 4.1. Is the sampling strategy relevant to address the research question? | 4.2. Is the sample representative of the target population? | 4.3. Are the measurements appropriate? | 4.4. Is the risk of nonresponse bias low? | 4.5. Is the statistical analysis appropriate to answer the research question? |
| 1 | Weintraub et al. (2016) | Yes | Yes | Yes | Yes | Yes | Yes | Yes |
| 2 | Contreras et al. (2016) | Yes | Yes | Yes | Yes | Yes | Yes | Yes |
| 3 | Waelde et al. (2018) | Yes | Yes | Yes | No | Yes | Yes | Yes |
