# Supplementary material for: Mental health interventions for climate change-induced disaster survivors in low- and middle-income countries (LMICs): A systematic review
Source: Glob Ment Health (Camb). 2026 Jun 15;13:e154. doi: 10.1017/gmh.2026.10249 (PMC13375834; doi:10.1017/gmh.2026.10249)
Supplement: Peer review history

# Reviewer report: Review: Mental health interventions for climate change-induced disaster survivors in low- and middle-income countries (LMICs): A systematic review — R0/PR2

**DOI:** 10.1017/gmh.2026.10249.pr2

**Authors:** Kiffer Card

**Affiliations:** Simon Fraser University, Canada

Abstract - Authors say studies cover specialist and non-specialist services, which is not very informative.

Abstract - The conclusion that there is a gap is not supported by the data of this review because it does not identify a gap it reviews studies of services.

Abstract - the comment about individual interventions feels a bit out of place without a sense of what range of non-individual supports might be otherwise offered (doesn’t post-disaster mental health support necessarily focus on individuals given reactions to such events are so individual-specific? I’m not aware of effective interventions for traumatic mental health supports that don’t focus on individuals, especially in a research context (which this review is limited to due to methodological choice).

Background - it’s a bit odd to note that 85% of population lives in LMIC, but only 80% of mental health problems there as this makes it seem they’re under-represented in mental health disparities…I would omit the distribution piece.

Background - fundamentally the question of why these services and supports are missing is not addressed or contextualized, which I think would be important to advocating for their integration into existing health systems.

Methods - please provide your search terms in a table so we can review these. It would help to describe how your search terms were generated and how they compare with other reviews in the four areas your review touches on.

Results - in the table can you report when the studies were conducted XX months or years after the disaster? This is relevant for understanding when these treatments are being administered compared to when the events took place.

Results - I think it’s worth clarifying/emphasizing that the types of studies typically captured in research tend to focus on the types of interventions you surfaced. It’s no surprise the more structural and community pieces are missing because when these interventions occur, they’re rarely published on. I would avoid asserting that absence from western academic journals implies that efforts for community resilience etc are not happening.

# Reviewer report: Review: Mental health interventions for climate change-induced disaster survivors in low- and middle-income countries (LMICs): A systematic review — R0/PR3

**DOI:** 10.1017/gmh.2026.10249.pr3

**Authors:** Cate Woods

**Affiliations:** University of Canterbury, New Zealand

This study reviews the existing literature evaluating mental health interventions for climate-induced disasters in lower- and middle-income countries. This is an important area of research, given that relatively few studies have been conducted in these settings. The review utilizes a comprehensive search strategy and robust screening methods to identify all available studies on this topic. However, the review has several limitations, including a lack of formal quality assessment reporting and some potential conceptual issues regarding the use of the IASC framework.

Introduction:

Pg. 2; lines 27-28: It may be worth clarifying that while disasters (including climate-induced disasters) are associated with mental health consequences, research also indicates that most disaster-exposed people do not develop ongoing mental health issues (i.e., exhibit a resilient trajectory of post-disaster functioning).

Method:

Pg. 3; line 59: What is the rationale for excluding studies published prior to the year 2000? Valuable information may be contained in studies published outside of this date range.

Pg. 5: According to the PRISMA flow diagram, two studies were excluded after quality appraisal, which was conducted using the MMAT. I cannot find any mention of a rationale or protocol for excluding studies on the basis of pre-determined quality criteria. Additionally, this did not form part of the pre-registered PROSPERO protocol. Unless there were very serious methodological concerns for these two studies, I wonder if it would be more appropriate to include them in the review and discuss their findings in the context of these methodological considerations.

Results:

Pg. 17, lines 14-15: I assume that the authors have categorized studies as RCTs, non-randomized, and quantitative descriptive studies as these correspond to distinct quality assessment criteria in the MMAT. 1) In Tables 1 and 2, some studies do not appear to be categorized this way (e.g., Table 1 notes there is one “quasi-experimental study” and Table 2 describes the Ede et al. study as a “pretest-posttest control group design). Using consistent categories will improve the clarity of the Results for readers. 2) I am not sure I agree with the categorization of some studies. For instance, the study by Aduriz et al. is described as a quantitative descriptive study, but as this study contains a comparative component (i.e., a pre-post comparison), this sounds more like a non-randomized study according to the MMAT classification.

Pg. 7; lines 47-48: I am not sure that this study is able to draw conclusions about the frequency of climate-induced disasters over time.

Pg 9; lines 11-12: The lack of studies reporting on government-led initiatives may reflect the lack of these initiatives in general, but it could also be the case that these initiatives have not been formally evaluated using empirical research methods.

Pg. 12; lines 5-11: The authors suggest that specialized and non-specialized support services are overrepresented, with relatively few studies evaluating basic services (i.e., Layer 4 interventions). The IASC framework contains four tiers of intervention, ranging from those intended to address the needs of the disaster-exposed population more broadly (i.e., the bottom tiers), to those intended for the minority of individuals who develop severe and ongoing disaster-related distress (i.e., the top tiers). Given that Layer 4 interventions are intended to address the needs of the disaster-exposed population more broadly, and given that most disaster-exposed people do not develop ongoing mental health issues, is it possible that few studies of Layer 4 interventions were identified because these types of initiatives are not usually intended to address mental health issues (e.g., PTSD, anxiety, depression)?

Pg. 19; lines 42-45: The authors note that there is some overlap between Layer 1 and 2 interventions, and that the current study distinguishes between the two based on the type of provider delivering the intervention. I am not sure I completely agree with this method of distinguishing interventions. For instance, Narrative Exposure Therapy (Crombach & Siehl, 2018) is classed as a Layer 2 intervention, even though this intervention was designed as a specialized intervention for PTSD.

The authors state that the quality of the included studies was assessed, but there is no discussion of the results of these quality assessments. Including a narrative synthesis of study quality would strengthen the review and has the potential to identify important methodological limitations in the area.

Discussion:

It may be worth noting in the limitations section that only one study was conducted in a low-income country.

Limitations of the review processes are discussed, but there is no discussion of the broad methodological limitations in this area, which would be facilitated by including the results of the quality assessment in the paper.

# Reviewer report: Review: Mental health interventions for climate change-induced disaster survivors in low- and middle-income countries (LMICs): A systematic review — R0/PR4

**DOI:** 10.1017/gmh.2026.10249.pr4

Review Article: Mental health interventions for climate change-induced disaster survivors in Low- and Middle-Income Countries (LMICs): A systematic review

General Comments: This is a well-written, timely, and deeply insightful study. This study offers a valuable systematic review of mental health interventions for climate disaster survivors in LMICs, highlighting significant gaps, particularly in community-based and systems-level care. It highlights the existence of comprehensive, evidence-based strategies. However, further details on the effectiveness of interventions and policy recommendations would enhance their practical relevance for policymakers and disaster management practitioners.

Background

1. The Background section effectively outlines the significant impact of climate change on low- and middle-income countries (LMICs), particularly through extreme weather events such as floods, cyclones, and droughts. (a) The authors may need specific examples and real-life case studies to illustrate their points. (b) Highlights mental health crises in disaster-prone regions like Bangladesh and the Philippines. (c) Emphasizes the urgency of addressing mental health.

2. While the connection between climate change and mental health is clearly established, the background does not fully explain the relevance of the Inter-Agency Standing Committee (IASC) framework in the context of climate-induced disasters. (a) Provides a foundation for study. (b) Details support levels for disaster survivors. (c) Enhances understanding of the framework’s importance.

3. The background section mentions the increasing frequency of climate-related disasters but lacks a detailed exploration of how this trend directly correlates with escalating mental health challenges in LMICs. The authors might provide more concrete evidence from global organizations regarding climate disasters. For example, (a) statistics from the WHO and IPCC indicate that mental health worsens due to the increasing frequency of climate disasters. (b) Evidence gaps highlight underexplored disaster types and regions.

4. The discussion on socioeconomic vulnerabilities is crucial but could be expanded to explore better how factors like poverty, poor healthcare infrastructure, and social inequality amplify the mental health impacts of climate-induced disasters. (a) Examining intersecting vulnerabilities. (b) Understanding challenges faced by disaster-affected populations.

5. Finally, while the background touches on global frameworks such as the UN Sustainable Development Goals (SDGs), it would benefit from further discussion on how these frameworks are integrated into local efforts. Specifically, how do local governments and NGOs adapt and apply these global frameworks to address the mental health needs of survivors of climate disasters? (a) Adapting global frameworks to meet climate disaster survivors' mental health needs. (b) Providing examples for a holistic view of international and local strategies.

Methodology: The methodology section provides a solid foundation for the systematic review, but several aspects could benefit from additional clarification and detail to enhance transparency and rigor.

1. The rationale for selecting the six databases (Medline, Embase, PsycINFO, etc.) is not explained. Why were these databases chosen over others, and how do they align with the research focus on mental health interventions in LMICs? The selection of specific databases, particularly those focusing on LMICs and the intersection of climate-induced disasters and mental health, as well as their exclusions, would provide valuable insights. (a) The author might focus on LMICs. (b) Intersection of climate-induced disasters and mental health. (c) Exclusion of other databases.

2. The study mentions following the PRISMA guidelines, but a rationale for this choice is not provided. Why were these guidelines selected, and how do they contribute to the rigor of the review? The PRISMA guidelines are crucial for enhancing the reliability and consistency of systematic reviews, thereby improving the transparency of the methodology and ensuring systematic and transparent processes, as explained in the summary. (a) The authors should enhance the reliability and consistency of systematic reviews. (b) Improve methodology transparency. (c) Ensuring that systematic and transparent processes are in place is crucial.

3. The search strategy is outlined, but there is no mention of whether language restrictions were applied. Were only English-language articles included, and if so, why? Language restrictions could clarify the exclusion of non-English articles and grey literature, allowing for a broader review scope and potential insights from non-peer-reviewed journals. The authors should clarify (a) the exclusion of non-English articles due to language restrictions. (b) Consideration of non-peer-reviewed or grey literature for a broader scope. (c) Value of insights from non-peer-reviewed journals.

3. The inclusion criteria (e.g., studies from 2000–2024) are outlined, but the methodology does not mention whether specific study designs or methodological quality criteria were required for inclusion. Were studies with methodological flaws excluded? Include study design details, methodological quality criteria, and risk of bias assessment for transparency and understanding of strengths and weaknesses in the selection process. There is a need to provide further details, including (a) specifics about the study design and methodological quality. (b) Prioritization of RCTs, observational studies, or qualitative research. (c) Risk of bias assessment. (d) Enhances transparency in the selection process.

4. The use of the Mixed Methods Appraisal Tool (MMAT) for quality appraisal is mentioned. But there’s no explanation of how study quality was assessed or how author discrepancies were resolved. More detailed information could enhance the study’s methodology, including the application of the MMAT and handling of author discrepancies. The authors should elaborate on (a) the details of the MMAT application and quality assessment criteria. (b) Assurance of consistency and reliability in data extraction. (c) Strengthening the review process through discussion and third-party review.

5. While the methodology mentions that two authors independently screened the studies, there is no explanation of how consistency was ensured during this process. Were any discrepancies resolved by a third reviewer or through consensus? The transparency of the review process can be enhanced by addressing discrepancies in study screening and data extraction, thereby ensuring reliability and consistency in the selection process. The authors should expand on (a) explaining the handling of discrepancies in study screening and data extraction. (b) Explain the involvement of the third reviewer. (c) Confirm the consensus reached after the discrepancy discussion. (d) Reassurance about the reliability and consistency of the study selection process.

6. The methodology mentions how data were extracted, but there is little detail about how the data were synthesized or analyzed. Was a narrative synthesis or meta-analysis conducted to summarize the findings? The data synthesis approach, which includes categorization, quantitative pooling, and qualitative synthesis, helps readers understand how study results were combined and analyzed, thereby identifying patterns or trends. However, the author should expand on the data synthesis approach. (a) Categorized studies by intervention type or mental health outcomes. (b) Conducted quantitative pooling or qualitative synthesis. (c) Clarified integration of results and identification of patterns or trends.

7. The methodology mentions that only studies published in English were included and that no studies from high-income countries were considered. Are there other potential limitations that affect the generalizability of the findings? The review’s scope could be improved by addressing methodological limitations, such as the exclusion of non-English studies and grey literature, and considering the applicability of the findings to low-income countries. The authors should delve deeper into (a), specifically addressing methodology limitations such as the exclusion of non-English studies or grey literature. (b) Discuss the applicability of findings to high-income countries. (c) Discuss the transferability of interventions to Low-Income Countries (LMICs).

Results: While the Results section offers an extensive summary of the included studies, it could improve clarity and depth in several areas. Below are suggestions to strengthen the presentation and analysis of the findings.

1. The results section provides a summary of study characteristics but does not fully break down mental health outcomes based on disaster types (floods vs. typhoons). How do the outcomes differ between these disaster types? Presenting mental health outcomes separately for each disaster type, such as PTSD, depression, and anxiety, can enhance clarity and help identify trends. The authors may consider (a) presenting mental health outcomes separately for each disaster type. (b) Use a table or a figure to compare the prevalence of disorders. (c) Enhance clarity and identify trends.

2. The results mention that the most common mental health disorders are anxiety, depression, and PTSD, but the exact prevalence is only noted in percentages. Are there significant differences in the prevalence of these disorders across different regions or types of disasters? A detailed analysis of disorder prevalence by region and disaster type, along with a table listing cases, would provide a clearer understanding of the distribution of mental health conditions. The authors may consider these variables, such as (a) breakdown by region and disaster type. (b) Include a table listing cases. (c) Provide a precise distribution of mental health conditions.

3. The results provide an overview of various interventions, but they do not discuss their effectiveness in detail. Which interventions were most successful in alleviating mental health symptoms in survivors? The discussion could be improved by comparing the efficacy of various interventions, such as EMDR, CBT, and community-based support, and providing a table summarizing their reported effectiveness.

4. The studies are categorized according to the IASC framework (layers 1–4), but further clarification is needed on the distribution of studies across each layer. How do interventions map to these layers? A detailed breakdown of studies on different layers of support, along with a figure illustrating their distribution, could provide a more comprehensive understanding of the concentration of interventions.

5. The results note that six studies reported NGO involvement in mental health interventions, while only one study mentioned government-led interventions. How does this affect the effectiveness of the interventions? The authors may provide a comprehensive analysis of the effectiveness of NGOs and government interventions in mental health care. (a) NGOs vs. Government Involvement in Mental Health Interventions. (b) NGOs' interventions are more effective than those led by the government. (c) The effectiveness of mental health interventions varies based on the type of service provider.

6. The results present a lot of data, but the key findings are not immediately apparent. What are the most critical takeaways from this review in terms of gaps in mental health interventions for climate disaster survivors? The results section could benefit from a summary paragraph or a key findings box that outlines the key findings, gaps in interventions, under-researched regions, and the most effective interventions. Such as (a) highlight main takeaways. (b) Highlight gaps in interventions. (c) Highlight under-researched regions. (d) Discuss the most effective interventions.

Discussion: The discussion section of this study presents a comprehensive analysis of mental health interventions in disaster response within low- and middle-income countries (LMICs). It highlights several areas that could benefit from further elaboration to enhance the depth and practical relevance of the findings. (a) The authors should make more direct comparisons between their conclusions and existing studies, particularly those focused on similar populations or disaster types. (b) Expand on the barriers to scaling up community-based and systems-level interventions, including cultural, logistical, and financial challenges. (c) The authors should provide actionable policy recommendations, such as integrating mental health services into national emergency response plans or investing in the training of community workers to deliver mental health support during disasters. (d) Discuss whether minimal interventions (e.g., psychoeducation, mindfulness) are as effective as more intensive therapies (e.g., trauma-focused psychotherapy). (e) Outline specific areas where research is lacking, such as particular disaster types or populations that require more focused study.

# Reviewer report: Review: Mental health interventions for climate change-induced disaster survivors in low- and middle-income countries (LMICs): A systematic review — R0/PR5

**DOI:** 10.1017/gmh.2026.10249.pr5

**Authors:** Usoro Akpan

**Affiliations:** Public Health, University of Warwick, United Kingdom of Great Britain and Northern Ireland

This manuscript is a systematic review examining mental health interventions for climate change-induced disaster survivors in LMICs represents a well and comprehensively written work.

The manuscript successfully addresses a critical knowledge gap by mapping existing interventions against the Inter-Agency Standing Committee (IASC) framework, revealing a concerning concentration of services in individual-level care (Layers 1 and 2) rather than community-based and systems-level approaches.

However justify for the 2000-2024 timeframe and more criteria for distinguishing between IASC framework layers.

The discussion could be enhanced by addressing limitations more thoroughly, particularly the exclusion of other climate-induced disasters like droughts and heatwaves, and by providing more specific recommendations for implementing community-based interventions in resource-constrained settings.

# Reviewer report: Review: Mental health interventions for climate change-induced disaster survivors in low- and middle-income countries (LMICs): A systematic review — R0/PR6

**DOI:** 10.1017/gmh.2026.10249.pr6

This is a systematic review of mental health interventions to support individuals affected by natural disasters in low- and middle-income countries. This is an important topic given the increasing number of natural disasters in the context of climate change and the disproportionate mental health and social burden that individuals face in low-resource settings. The authors searched six databases and identified fourteen original papers.

Unfortunately, there are limitations that the authors are recommended to address prior to its publication.

1. My biggest concern is that the introduction is lacking considerations of existing review articles on a similar topic, e.g., “Mental health and psychosocial interventions in the context of climate change” by Xue et al 2024, as well as numerous previous systematic reviews, meta-analyses on psychosocial interventions for individuals exposed to natural disasters (e.g. Kip et al 2024, Brown et al 2017, İkican 2023), including a Cochrane review on “Psychological and social interventions for the prevention of mental disorders in people living in low‐ and middle‐income countries affected by humanitarian crises” by Papola et al 2020. The current review has not addressed potentially significant thematic overlaps with previous work or justified the need for a new review.

2. Methods: The authors are recommended to clarify the methods. It is for example mentioned that Google Scholar is used and this identified 100 hits - this is unclear why this method is used when the authors excluded grey literature or how this outcome was achieved. The inclusion and exclusion criteria are recommended to be significantly expanded - how did the authors determine if a disaster is “climate change induced” versus any type of environemental hazard or natural disaster? How are mental health and associated interventions defined (e.g., did the authors involve interventions for individuals meeting criteria for a mental disorder, or could this be interventions to prevent the onset of a mental disorder, an intervention aimed to improve mental wellbeing of any individuals exposed to disasters?). There are multiple interventions identified in previous reviews that are not included in this current review, and it raises question for the methods authors implemented.

3. The Results, Intervention Analysis and Discussion sections would benefit from significant restructuring as the authors made frequent interpretations of findings in the Results section. The individual studies identified could be summarized and described further, including the outcomes if the interventions. The quality appraisal outcomes were not described. The main finding of the review, which was mentioned in all above sections, is that there are more individual-level interventions than more upstream interventions - a finding that was identified in previous reviews. The authors are recommended to consider other types of nuanced analyses, including regional differences, timing of the interventions, mental health conditions or wellbeing states being targeted, and implementation models.

# Editor report: Recommendation: Mental health interventions for climate change-induced disaster survivors in low- and middle-income countries (LMICs): A systematic review — R0/PR7

**DOI:** 10.1017/gmh.2026.10249.pr7

**Affiliations:** Faculty of Business, Technology, and Education , Ilia State University, Georgia

No accompanying comment.

# Editor report: Decision: Mental health interventions for climate change-induced disaster survivors in low- and middle-income countries (LMICs): A systematic review — R0/PR8

**DOI:** 10.1017/gmh.2026.10249.pr8

**Affiliations:** Johns Hopkins University Bloomberg School of Public Health, United States

No accompanying comment.

# Reviewer report: Review: Mental health interventions for climate change-induced disaster survivors in low- and middle-income countries (LMICs): A systematic review — R1/PR10

**DOI:** 10.1017/gmh.2026.10249.pr10

**Authors:** Usoro Akpan

**Affiliations:** Public Health, University of Warwick, United Kingdom of Great Britain and Northern Ireland

This systematic review addresses a critical gap by synthesising mental health interventions for climate disaster survivors in LMICs using the IASC MHPSS framework. The methodology is sound: PRISMA-compliant protocol registered on PROSPERO (CRD42023456582), comprehensive six-database search screening 8,228 records, dual independent review with MMAT quality appraisal, yielding 14 final studies. The key finding reveals severe imbalance in intervention delivery, 79% of studies focused on individual-level clinical care (Layers 1-2: specialized/focused non-specialized services) while community support (Layer 3: 2 studies) and basic service integration (Layer 4: 1 study) remain critically underrepresented. The IASC framework mapping effectively demonstrates that NGOs delivered most interventions (6 studies) versus only one government-led programme, highlighting systemic gaps in national disaster response.

Revisions needed:

(1) Resolve the PRISMA flowchart discrepancy, it shows 16 full-text articles assessed leading to 14 final studies, but methods state “two studies excluded” during quality appraisal while maintaining n=14; clarify whether exclusions occurred during eligibility or quality assessment.

(2) Address conceptual overlap between Layers 1-2 more explicitly, particularly regarding complex interventions like Narrative Exposure Therapy delivered by lay workers.

Overall, a valuable contribution providing the first climate-specific MHPSS synthesis with actionable policy guidance for scaling disaster mental health interventions in LMICs.

# Reviewer report: Review: Mental health interventions for climate change-induced disaster survivors in low- and middle-income countries (LMICs): A systematic review — R1/PR11

**DOI:** 10.1017/gmh.2026.10249.pr11

Dear Editor,

Thank you for sending the revised manuscript, “Mental health interventions for climate change-induced disaster survivors in Low- and Middle-Income Countries (LMICs): A systematic review” (GMH-2025-0255.R1). I have carefully reviewed the revised manuscript and am pleased to confirm that the authors have thoroughly and thoughtfully addressed all of my previous comments and suggestions. I do not have any further revisions to request. Thank you for the opportunity to review this important study.

# Reviewer report: Review: Mental health interventions for climate change-induced disaster survivors in low- and middle-income countries (LMICs): A systematic review — R1/PR12

**DOI:** 10.1017/gmh.2026.10249.pr12

**Authors:** Cate Woods

**Affiliations:** University of Canterbury, New Zealand

The revised manuscript is improved by a more detailed description of the methodology and a clearer explanation of the findings in the context of the IASC model. There are several points (detailed below) that would further improve the clarity of the manuscript.

Abstract:

Pg. 30, lines 12-13: What is a “non-licensed mental health professional”? All mental health professionals (e.g., psychiatrists, psychologists, counsellors, mental health nurses etc.) are generally licensed under a professional body.

Introduction:

Pg. 32, lines 56-58: The point “Real-world examples illustrate the scale of this burden: Bangladesh’s 2022 monsoon floods 57 displaced over 7 million people, while Typhoon Haiyan in the Philippines caused extensive 58 destruction and long-lasting psychosocial impacts across affected communities” should be accompanied by citations.

Method:

Pg. 37, lines 245 to 247: This is slightly confusing. The beginning of this paragraph states that there were 14 studies reviewed, which is consistent with the rest of the manuscript and the PRISMA diagram. However, the end of this paragraph talks about two studies being excluded. Why are these two studies mentioned here? The PRISMA diagram indicates that a much larger number of studies were excluded for various reasons, what is special about these two that they need to be mentioned at the end of this paragraph?

Pg. 38, lines 255-258: The point “Finally, several methodological limitations must be acknowledged: exclusion of non-English and 256 grey literature, limited representation of low-income countries, variability in study design quality, 257 and the predominance of short-term outcomes limit the generalisability of findings. These constraints 258 highlight the need for more rigorous, longitudinal, and locally grounded research in LMICs.”, while important, should be included in the Discussion, not in the Method section.

When describing the IASC model, I wonder if it would be helpful to provide some examples of mental health interventions at each level of the model.

Results:

Pg. 43, lines 44-45: The point “Among these, four studies reported RCT designs, which 44 generally demonstrated stronger methodological rigour, whereas many observational studies suffered 45 from small sample sizes and short follow-up periods” seems like it belongs in the section focused on results of the quality assessments, rather than here.

Pg. 44, lines 56-58: There is mention of effect sizes here, but I cannot find any specific effect sizes from studies reported in the paper. It would be helpful to include reported effect size estimates in Table 2, alongside a summary of each study’s findings. Doing so will allow readers to better understand the magnitude of treatment effects.

Pg. 44, lines 65-66: What constitutes “moderate reductions”? Is this based on established thresholds for effect size interpretation (e.g., there are established thresholds for interpreting standardised mean differences such as Cohen’s d), or is this more of a subjective judgement by the authors?

Overall, I think there needs to be more of an in-depth synthesis of the study findings themselves (i.e., the study results). The focus is currently more on what interventions were evaluated across studies.

Pg. 44, lines 76-77: “The results from this study indicate that floods were most prevalent in lower-middle income countries (64%)”. This point does not seem relevant here.

Pg. 54, line 202: “A comparative summary of effectiveness is presented in Table 3.” Table 3 does not seem to contain any information that would allow a reader to compare the effectiveness of the interventions included in the review. See also my point above re: including reported effect sizes in Table 3.

I still do not see a detailed section describing the results of the formal quality assessment process. I see there is a table in the Appendix, which is good, but I think a summary of these findings in the main body of the Results would aid interpretation. Perhaps colour coding the cells in the Appendix table would also help readers identify patterns in the quality of the included studies.

# Editor report: Recommendation: Mental health interventions for climate change-induced disaster survivors in low- and middle-income countries (LMICs): A systematic review — R1/PR13

**DOI:** 10.1017/gmh.2026.10249.pr13

**Affiliations:** Faculty of Business, Technology, and Education , Ilia State University, Georgia

No accompanying comment.

# Editor report: Decision: Mental health interventions for climate change-induced disaster survivors in low- and middle-income countries (LMICs): A systematic review — R1/PR14

**DOI:** 10.1017/gmh.2026.10249.pr14

**Affiliations:** Johns Hopkins University Bloomberg School of Public Health, United States

No accompanying comment.

# Reviewer report: Review: Mental health interventions for climate change-induced disaster survivors in low- and middle-income countries (LMICs): A systematic review — R2/PR16

**DOI:** 10.1017/gmh.2026.10249.pr16

**Authors:** Usoro Akpan

**Affiliations:** Public Health, University of Warwick, United Kingdom of Great Britain and Northern Ireland

This systematic review addresses a highly relevant and timely topic — mental health and psychosocial support (MHPSS) interventions for climate disaster survivors in LMICs. It applies the IASC framework as an organising lens, which is a useful and policy-relevant approach. The study follows PRISMA guidelines, registers a protocol on PROSPERO, uses six databases, employs the MMAT for quality appraisal, and provides clear tabulation of included studies. These are genuine strengths. However, this manuscript still contains significant issues that must be resolved before acceptance

Concerns

1. Inclusion of Upper-Middle Income Countries (UMICs)

Four of the 14 included studies (28.6%) were conducted in upper-middle income countries (UMICs — Argentina, China, Peru, and Tuvalu), yet the review is framed as examining LMICs. This is a significant conceptual inconsistency. The title, abstract, and conclusions claim the findings apply to LMICs, but a sizable proportion of the evidence base comes from a different income category. The authors should either revise the framing to accurately reflect that the review covers LMICs and UMICs, or justify why UMIC evidence is pertinent to LMIC-specific conclusions.

2. Very Small Final Corpus (n=14)

From 8,228 records screened, only 14 studies were retained — a yield of 0.17%. While the authors explain this through the strict definition of climate-induced disasters and LMIC focus, it raises questions about whether the restrictive eligibility criteria may have inadvertently excluded relevant studies. A brief reflection is needed on whether specific eligibility decisions materially affected the yield, and how this limitations the review’s conclusions.

3. Narrow Disaster Type Coverage

All 14 studies focus exclusively on floods (n=11) and typhoons (n=3). No studies on droughts, heatwaves, wildfires, or other climate disasters were identified, despite these being included in the search terms. While the authors acknowledge this gap in the limitations, the conclusions and policy implications are derived entirely from flood and typhoon evidence. The authors should be more explicit about the risks of generalising these findings to MHPSS responses for other climate hazards, which may have substantially different mental health profiles.

4. Weak Conclusion Section

The conclusion section (a single paragraph) is notably brief and does not synthesise the key findings in a meaningful way. It largely restates the study’s aims rather than summarising what was found and what it means. Given the richness of the discussion, the conclusion should clearly restate the most important findings and what they collectively imply for practice and future research.

# Reviewer report: Review: Mental health interventions for climate change-induced disaster survivors in low- and middle-income countries (LMICs): A systematic review — R2/PR17

**DOI:** 10.1017/gmh.2026.10249.pr17

**Authors:** Cate Woods

**Affiliations:** University of Canterbury, New Zealand

I am satisfied that all of my comments/queries have been sufficiently addressed by the authors.

# Editor report: Recommendation: Mental health interventions for climate change-induced disaster survivors in low- and middle-income countries (LMICs): A systematic review — R2/PR18

**DOI:** 10.1017/gmh.2026.10249.pr18

**Affiliations:** Faculty of Business, Technology, and Education , Ilia State University, Georgia

No accompanying comment.

# Editor report: Decision: Mental health interventions for climate change-induced disaster survivors in low- and middle-income countries (LMICs): A systematic review — R2/PR19

**DOI:** 10.1017/gmh.2026.10249.pr19

**Affiliations:** Johns Hopkins University Bloomberg School of Public Health, United States

No accompanying comment.

# Editor report: Recommendation: Mental health interventions for climate change-induced disaster survivors in low- and middle-income countries (LMICs): A systematic review — R3/PR21

**DOI:** 10.1017/gmh.2026.10249.pr21

**Affiliations:** Faculty of Business, Technology, and Education , Ilia State University, Georgia

No accompanying comment.

# Editor report: Decision: Mental health interventions for climate change-induced disaster survivors in low- and middle-income countries (LMICs): A systematic review — R3/PR22

**DOI:** 10.1017/gmh.2026.10249.pr22

**Affiliations:** Johns Hopkins University Bloomberg School of Public Health, United States

No accompanying comment.
